# Supplementary material for: Natural variation in the zinc-finger-encoding exon of Prdm9 affects hybrid sterility phenotypes in mice
Source: Genetics. 2024 Jan 13;226(3):iyae004. doi: 10.1093/genetics/iyae004 (PMC10917509; doi:10.1093/genetics/iyae004)
Supplement: iyae004_Supplementary_Data [file iyae004_supplementary_data.zip › Figure_S2_GENETICS-2023-306660.pdf]

Mus musculus

alpha-helix

	Name	ZNF Score						-8			-5				-1	1		3		5	6			9				13				COLOR		
			P	Y	V	C	R	E	C	G	R	G	F	T	Q	K	S	D	L	I	Q	H	Q	R	T	H	T	R	E	K	*	Q	D	Q
**First repeats**	MM_01	11.7	S	S	I	E	R	Q	C	G	Q	Y	F	S	D	K	S	N	V	N	E	H	Q	K	T	H	T	G	E	K		D	N	E
	MM_t	fail	S	S	I	E	-	-	-	G	Q	Y	F	S	D	K	S	N	V	N	E	H	Q	K	T	H	T	G	E	K		D	N	E
**Internal repeats**	MM_02	33.1	P	Y	V	C	R	E	C	G	R	G	F	T	A	K	S	D	L	I	Q	H	Q	R	T	H	T	G	E	K		A	D	Q
	MM_03	35.3	P	Y	V	C	R	E	C	G	R	G	F	T	A	K	S	N	L	I	K	H	Q	R	T	H	T	G	E	K		A	N	K
	MM_04A	33.0	P	Y	V	C	R	E	C	G	W	G	F	T	A	K	S	N	L	I	Q	H	Q	R	T	H	T	G	E	K		A	N	Q
	MM_04B	32.3	P	Y	V	C	R	E	C	G	R	G	F	I	A	K	S	N	L	I	Q	H	Q	R	T	H	T	G	E	K		A	N	Q
	MM_05A	32.5	P	Y	V	C	R	E	C	G	R	G	F	T	A	K	S	S	L	V	Q	H	Q	R	T	H	T	G	E	K		A	S	Q
	MM_05B	33.0	P	Y	V	C	R	G	C	G	R	G	F	T	A	K	S	S	L	I	Q	H	Q	R	T	H	T	G	E	K		A	S	Q
	MM_06	33.9	P	Y	V	C	R	E	C	G	R	G	F	T	A	K	S	S	L	I	K	H	Q	R	T	H	T	G	E	K		A	S	K
	MM_07	30.7	P	Y	V	C	R	E	C	G	R	G	F	T	A	K	S	V	L	I	Q	H	Q	R	T	H	T	G	E	K		A	V	Q
	MM_08	35.5	P	Y	V	C	R	E	C	G	R	G	F	T	E	K	S	N	L	I	Q	H	Q	R	T	H	T	G	E	K		E	N	Q
	MM_09	36.3	P	Y	V	C	R	E	C	G	R	G	F	T	E	K	S	N	L	I	K	H	Q	R	T	H	T	G	E	K		E	N	K
	MM_10	34.9	P	Y	V	C	R	E	C	G	R	G	F	T	E	K	S	S	L	I	K	H	Q	R	T	H	T	G	E	K		E	S	K
	MM_11	37.4	P	Y	V	C	R	E	C	G	R	G	F	T	Q	K	S	D	L	I	K	H	Q	R	T	H	T	G	E	K		Q	D	K
	MM_12A	36.3	P	Y	V	C	R	E	C	G	R	G	F	T	Q	K	S	D	L	I	Q	H	Q	R	T	H	T	G	E	K		Q	D	Q
	MM_13A	36.2	P	Y	V	C	R	E	C	G	R	G	F	T	Q	K	S	H	L	I	Q	H	Q	R	T	H	T	G	E	K		Q	H	Q
	MM_13B	35.9	P	Y	V	C	R	E	C	G	R	G	F	T	Q	N	S	H	L	I	Q	H	Q	R	T	H	T	G	E	K		Q	H	Q
	MM_14A	36.6	P	Y	V	C	R	E	C	G	R	G	F	T	Q	N	S	H	L	I	K	H	Q	R	T	H	T	G	E	K		Q	H	K
	MM_14B	36.0	P	Y	V	C	R	E	C	G	W	G	F	T	Q	N	S	H	L	I	K	H	Q	R	T	H	T	G	E	K		Q	H	K
	MM_15A	37.8	P	Y	V	C	R	E	C	G	R	G	F	T	Q	K	S	N	L	I	Q	H	Q	R	T	H	T	G	E	K		Q	N	Q
	MM_16	39.3	P	Y	V	C	R	E	C	G	R	G	F	T	Q	K	S	N	L	I	R	H	Q	R	T	H	T	G	E	K		Q	N	R
	MM_17A	38.6	P	Y	V	C	R	E	C	G	R	G	F	T	Q	K	S	N	L	I	K	H	Q	R	T	H	T	G	E	K		Q	N	K
	MM_17B	37.5	P	Y	V	C	R	E	C	G	W	G	F	T	Q	K	S	N	L	I	K	H	Q	R	T	H	T	G	E	K		Q	N	K
	MM_18	35.0	P	Y	V	C	R	E	C	G	R	G	F	T	Q	K	S	V	L	I	K	H	Q	R	T	H	T	G	E	K		Q	V	K
	MM_19	34.2	P	Y	V	C	R	E	C	G	R	G	F	T	Q	K	S	V	L	I	Q	H	Q	R	T	H	T	G	E	K		Q	V	Q
	MM_20	37.4	P	Y	V	C	R	E	C	G	R	G	F	T	T	K	S	D	L	I	K	H	Q	R	T	H	T	G	E	K		T	D	K
	MM_21	35.9	P	Y	V	C	R	E	C	G	R	G	F	T	V	K	S	N	L	I	Q	H	Q	R	T	H	T	G	E	K		V	N	Q
	MM_22	35.3	P	Y	V	C	R	E	C	G	R	G	F	T	V	K	S	S	L	I	K	H	Q	R	T	H	T	G	E	K		V	S	K
	MM_23	33.0	P	Y	V	C	R	E	C	G	R	G	F	T	V	K	S	V	L	I	K	H	Q	R	T	H	T	G	E	K		V	V	K
	MM_24	32.1	P	Y	V	C	R	E	C	G	R	G	F	T	V	K	S	V	L	I	Q	H	Q	R	T	H	T	G	E	K		V	V	Q
**Last repeat**	MM_12B	36.6	P	Y	V	C	R	E	C	G	R	G	F	T	Q	K	S	D	L	I	Q	H	Q	R	T	H	T	R	E	K	*	Q	D	Q
	MM_12C	35.2	P	Y	V	C	R	E	C	G	W	G	F	T	Q	K	S	D	L	I	Q	H	Q	R	T	H	T	R	E	K	*	Q	D	Q
	MM_15B	37.8	P	Y	V	C	R	E	C	G	R	G	F	T	Q	K	S	N	L	I	Q	H	Q	R	T	H	T	R	E	K	*	Q	N	Q
