# Supplementary material for: Natural variation in the zinc-finger-encoding exon of Prdm9 affects hybrid sterility phenotypes in mice
Source: Genetics. 2024 Jan 13;226(3):iyae004. doi: 10.1093/genetics/iyae004 (PMC10917509; doi:10.1093/genetics/iyae004)
Supplement: iyae004_Supplementary_Data [file iyae004_supplementary_data.zip › Figure_S3_GENETICS-2023-306660.pdf]

Homo sapiens sapiens transgene

alpha-helix

	Name	ZNF Score									-5				-1	1		3		5	6			9	10			13					
First repeats	HS_01	11.0	K	V	K	Y	G	E	C	G	Q	G	F	S	V	K	S	D	V	I	T	H	Q	R	T	H	T	G	E	K	V	D	T
	transgene	11.0	S	S	I	E	R	Q	C	G	Q	Y	F	S	D	K	S	N	V	N	E	H	Q	K	T	H	T	G	E	K	D	N	E
Internal repeats	HS_02	36.6	P	Y	V	C	R	E	C	G	R	G	F	R	D	K	S	H	L	L	R	H	Q	R	T	H	T	G	E	K	D	H	R
	HS_03	36.8	P	Y	V	C	R	E	C	G	R	G	F	R	D	K	S	N	L	L	S	H	Q	R	T	H	T	G	E	K	D	N	S
	HS_04A	36.6	P	Y	V	C	R	E	C	G	R	G	F	S	N	K	S	H	L	L	R	H	Q	R	T	H	T	G	E	K	N	H	R
	HS_04B	35.9	P	Y	V	C	R	E	C	G	R	G	F	R	N	K	S	H	L	L	R	H	Q	R	T	H	T	G	E	K	N	H	R
	HS_05	33.1	P	Y	V	C	R	E	C	G	R	G	F	S	R	Q	S	V	L	L	T	H	Q	R	R	H	T	G	E	K	R	V	T
	HS_06	33.1	P	Y	V	C	R	E	C	G	R	G	F	S	W	K	S	H	L	L	I	H	Q	R	I	H	T	G	E	K	W	H	I
	HS_07	31.3	P	Y	V	C	R	E	C	G	R	G	F	S	W	Q	S	V	L	L	T	H	Q	R	T	H	T	G	E	K	W	V	T
Last repeat	HS_08	32.0	P	Y	V	C	R	E	C	G	R	G	F	S	D	R	S	S	L	C	Y	H	Q	R	T	H	T	G	E	K	D	S	Y
	stop/end	fail	P	Y	V	C	R	E	D	E	*																						
