# Supplementary material for: Natural variation in the zinc-finger-encoding exon of Prdm9 affects hybrid sterility phenotypes in mice
Source: Genetics. 2024 Jan 13;226(3):iyae004. doi: 10.1093/genetics/iyae004 (PMC10917509; doi:10.1093/genetics/iyae004)
Supplement: iyae004_Supplementary_Data [file iyae004_supplementary_data.zip › Figure_S5_GENETICS-2023-306660.pdf]

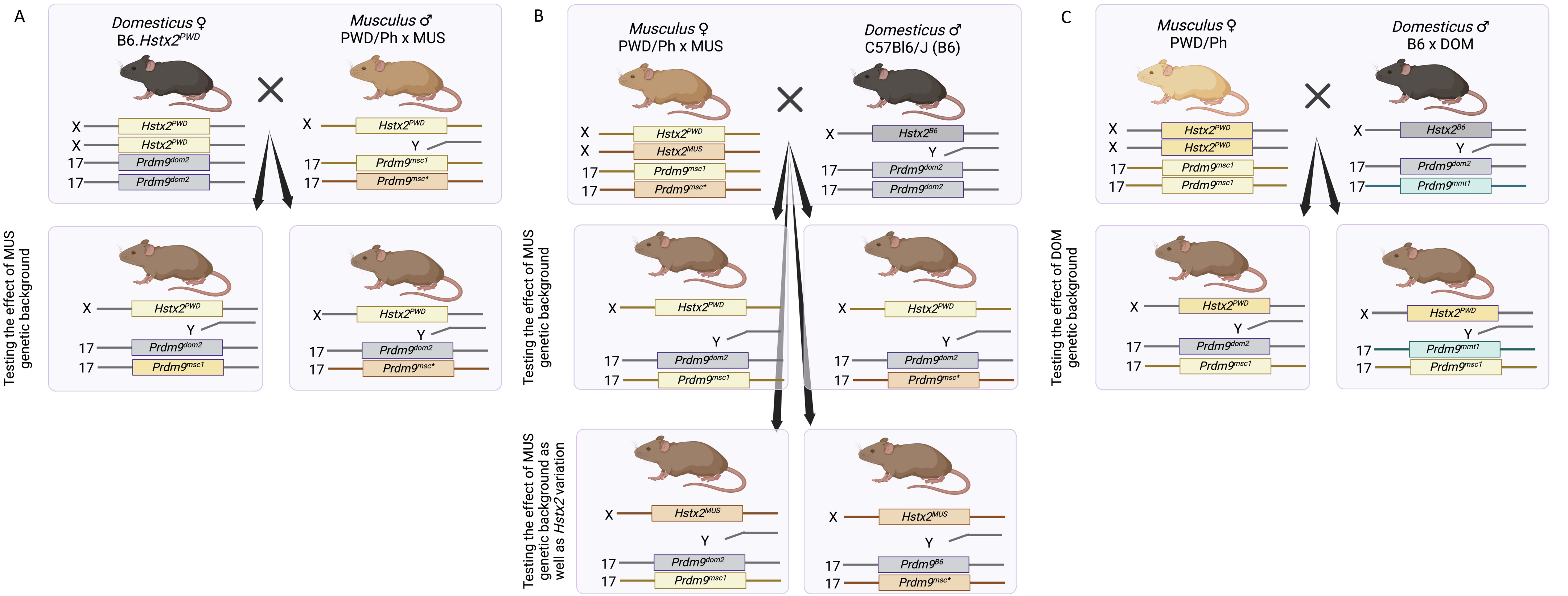

Figure S 9 Scheme of intersubspecific crosses and genotypes for testing the role of the genetic background . (A) B6.*Hstx2*^{PWD} females crossed to (PWD/Ph x MUS) intraspecific male hybrids (B) (PWD/Ph x MUS) hybrid females crossed to B6.*Hstx2*^{PWD} males, their offspring in the top panel, posses *Hstx2*^{PWD} , those in the lower panel inherited an unknown *Hstx2*^{MUS*}, whose effect was also assessed. PWD/Ph females crossed to (B6 x DOM) males produce interspecific hybrid males carrying either the 'sterility' *Prdm9*^{dom2} allele or *Prdm9*^{mmt1} the 'fertility' allele on mixed
