# Supplementary material for: Natural variation in the zinc-finger-encoding exon of Prdm9 affects hybrid sterility phenotypes in mice
Source: Genetics. 2024 Jan 13;226(3):iyae004. doi: 10.1093/genetics/iyae004 (PMC10917509; doi:10.1093/genetics/iyae004)
Supplement: iyae004_Supplementary_Data [file iyae004_supplementary_data.zip › Supplemental_Material_Legends_GENETICS-2023-306660.docx]

**Supplementary Data**

**
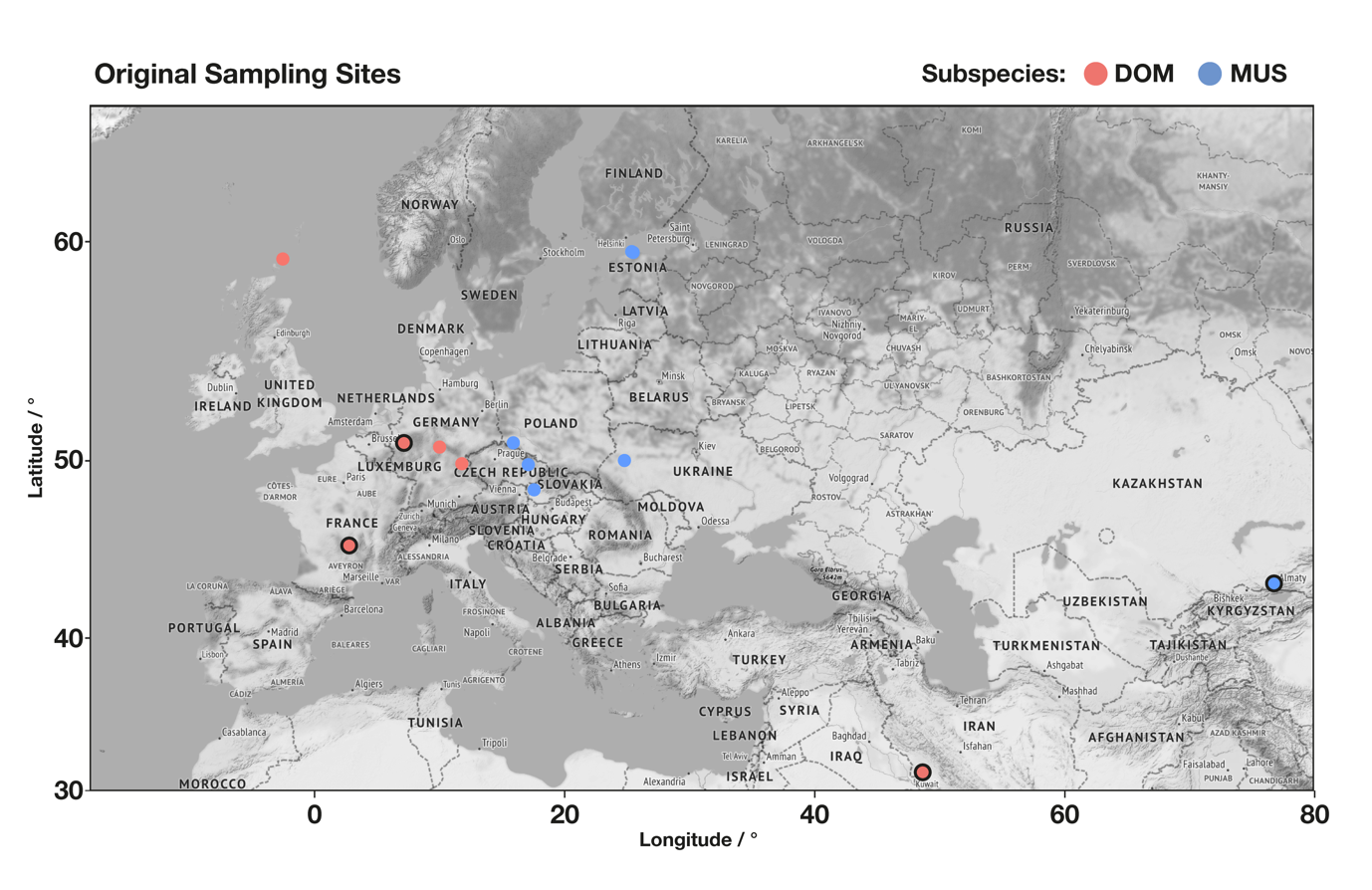
**

**Figure S1: Distribution of original** MUS (blue) and DOM (red) sampling sites. The framed circles represent mice evaluated for hybrid sterility in this study, while the plain circles are from ([Mukaj et al. 2020](#_ENREF_57))

**Figure S2: Types of Cysteine-2-Histidine-2 (C_2_H_2_) ZNFs** **found in PRDM9 MUS and DOM** in this study and in ([Mukaj et al. 2020](#_ENREF_57)). We highlighted the Cysteine and Histidine residues in yellow and shaded all variable amino acids in gray, distinguishing between amino acids by color. As amino acids in positions -1, 3, and 6 of the alpha-helix are those responsible for the DNA-binding specificity of a given ZNF (as shown in the cartoon of **Figure 8A)**, we used them as acronyms on the right. Boxes of the same color characterize the hypervariable positions of each C_2_H_2_ zinc finger in **Figure 1** and the phylogenetic analyses in **Figure 8**.

**Figure S3: Types of C_2_H_2_ zinc fingers found in “humanized PRDM9 variant “B”.** We highlighted the cysteine and Histidine residues in yellow and shaded variable amino acids in gray. Those in positions -1, 3, and 6 of the alpha-helix are responsible for DNA binding (as shown in **Figure 8A**), and we used them as acronyms on the right. Boxes of the same color characterize the hypervariable positions of each C_2_H_2_ zinc finger, as in **Figure 1**.


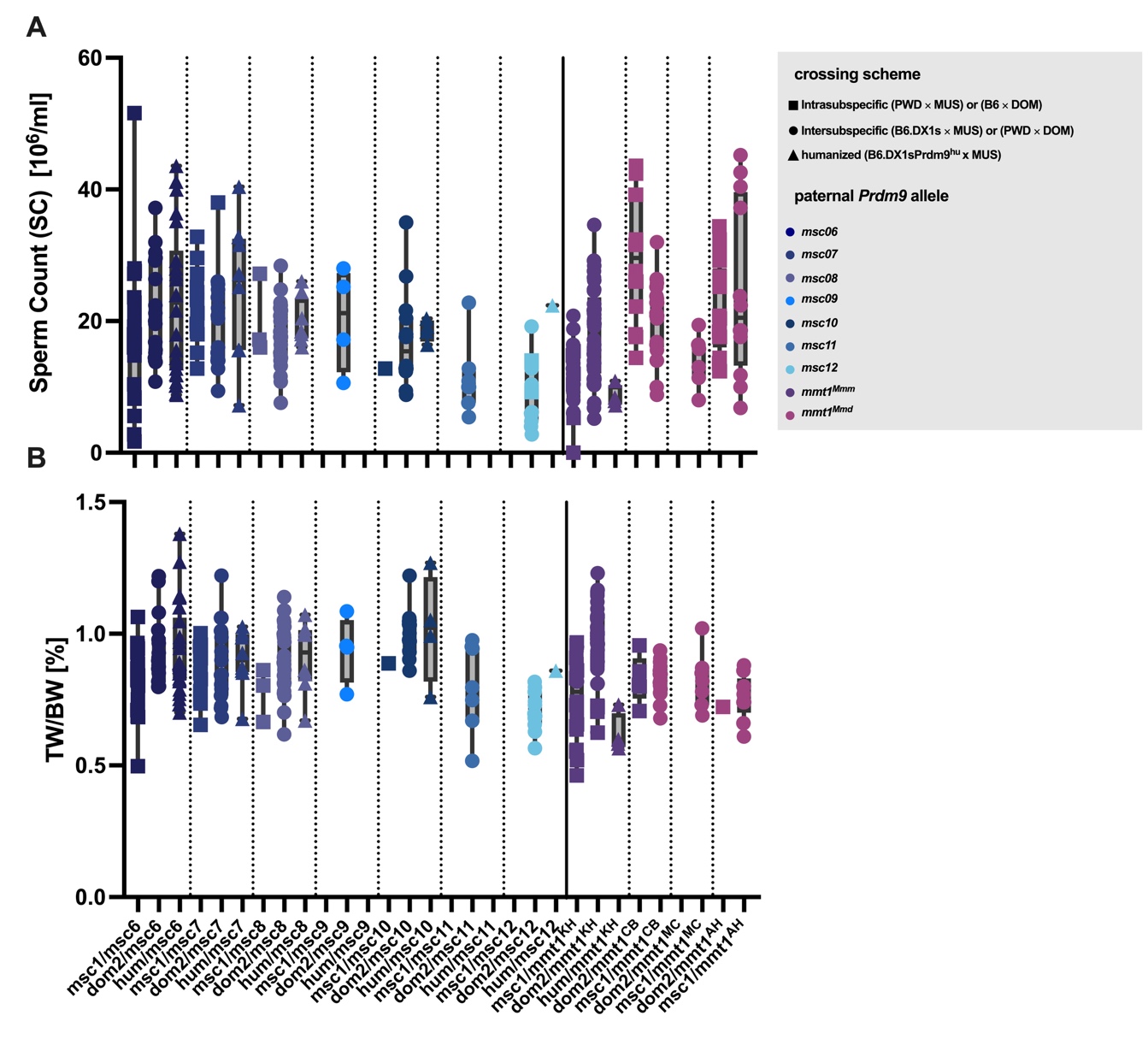


**Figure S4: Fertility parameters of intra- and intersubspecific F_1_-hybrids** **(A)** sperm count **(B)** paired testes weight normalized by body weight of intersubspecific and intrasubspecific mice. Colors distinguish paternal Prdm9 alleles, while the shape of the data points distinguishes crossing schemes. (Square data points) Intrasubspecific male F_1_ offspring of PWD females crossed to MUS males, or intrasubspecific B6 females crossed to DOM males. Two types of intersubspecific crosses were performed; MUS males were crossed to B6.DX1s females and wild DOM males were crossed to PWD females (circles as data points, also shown in **Figure 1**); for the MUS males, a third cross was performed where they were mated with B6.DX1s .*Prdm9^hu^* females (triangular data points). Since most males used as sires were heterozygous for different allelic combinations of *Prdm9,* data from several crosses were pooled by *Prdm9* genotype of the F_1_-hybrid offspring, and the significance of differences in fertility phenotypes was evaluated using ordinary one-way ANOVA with Bonferroni correction as before.

**Figure S5: Scheme of intersubspecific crosses and genotypes for testing the role of the genetic background**. **(A)** B6.*Hstx2^PWD^* females crossed to (PWD/Ph ×  wild MUS) intrasubpecific male hybrids. **(B)** (PWD/Ph ×  wild MUS) hybrid females crossed to B6.*Hstx2^PWD^* males, their offspring in the top panel, possess *Hstx2^PWD^. Those* in the lower panel inherited an unknown *Hstx2^MUS*^*, whose effect was also assessed. **(C)** PWD/Ph females crossed to (B6 × DOM) males produce intersubspecific hybrid males carrying either the ‘sterility’ *Prdm9^dom2^* allele or *Prdm9^mmt1,^* the ‘fertility’ allele on mixed DOM/B6 genetic background with MUS/DOM genomic heterozygosity. The image was created using BioRender.

**Figure S6: X-chromosomal haplotypes across the refined Hstx2 locus** from ([Lustyk et al. 2019](#_ENREF_54)) based on allelic variation at microsatellite markers SX65100, SR51, and SX69084 in laboratory and wild mice. On top, all genes and microRNAs annotated within this locus are shown in the mm38 reference genome. The AFLP/STR allele length (in bp) is shown on the bottom for different haplotypes.


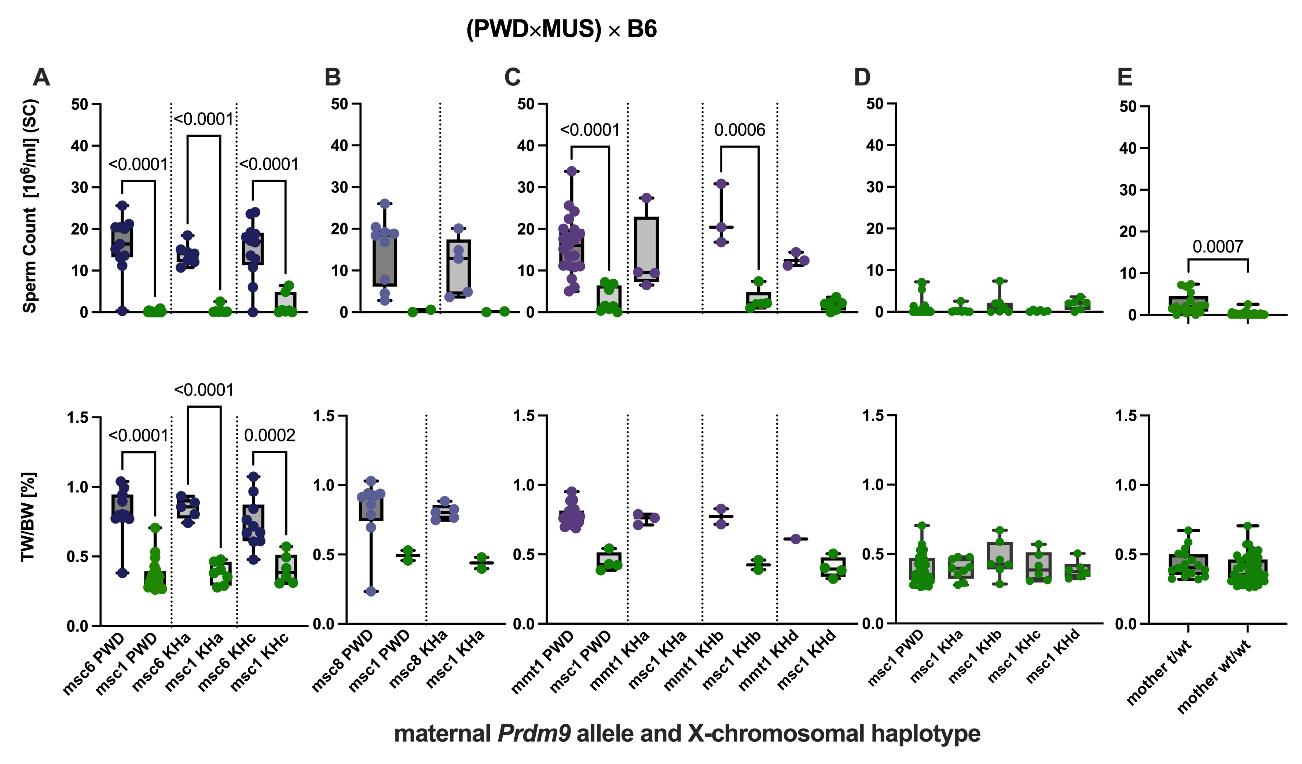


**Figure S7: Segregation analyses of maternal *Prdm9* genotype together with the variation of wild X-chromosomal haplotypes.** Intersubspecific F_1_ male offspring of intrasubspecific MUS hybrid females crossed to B6 males (as in **Figure 7B**) that inherited wild MUS X-chromosomal haplotypes. X-axes depict both the maternal *Prdm9* allele and wild MUS X-chromosomal haplotype, defined as shown in (**Figure S6**). Fertility parameters, sperm count (top), and paired testes weights (bottom) are shown, and the significance of differences in fertility phenotypes was evaluated as before. **(A-C)** Segregation analyses grouped by maternal *Prdm9* allele combination, **(A)** maternal *msc1* and *msc6* alleles, **(B)** maternal *msc1* and *msc8* alleles, **(C)** *msc1* and *mmt1^KH^* alleles. **(D-E)** Pooled data of hybrids from different mothers that all inherited the same *msc1/dom2* allelic combination **(D)** pooled by X-chromosomal haplotype, **(E)** pooled by whether mothers possessed t-haplotypes.

.


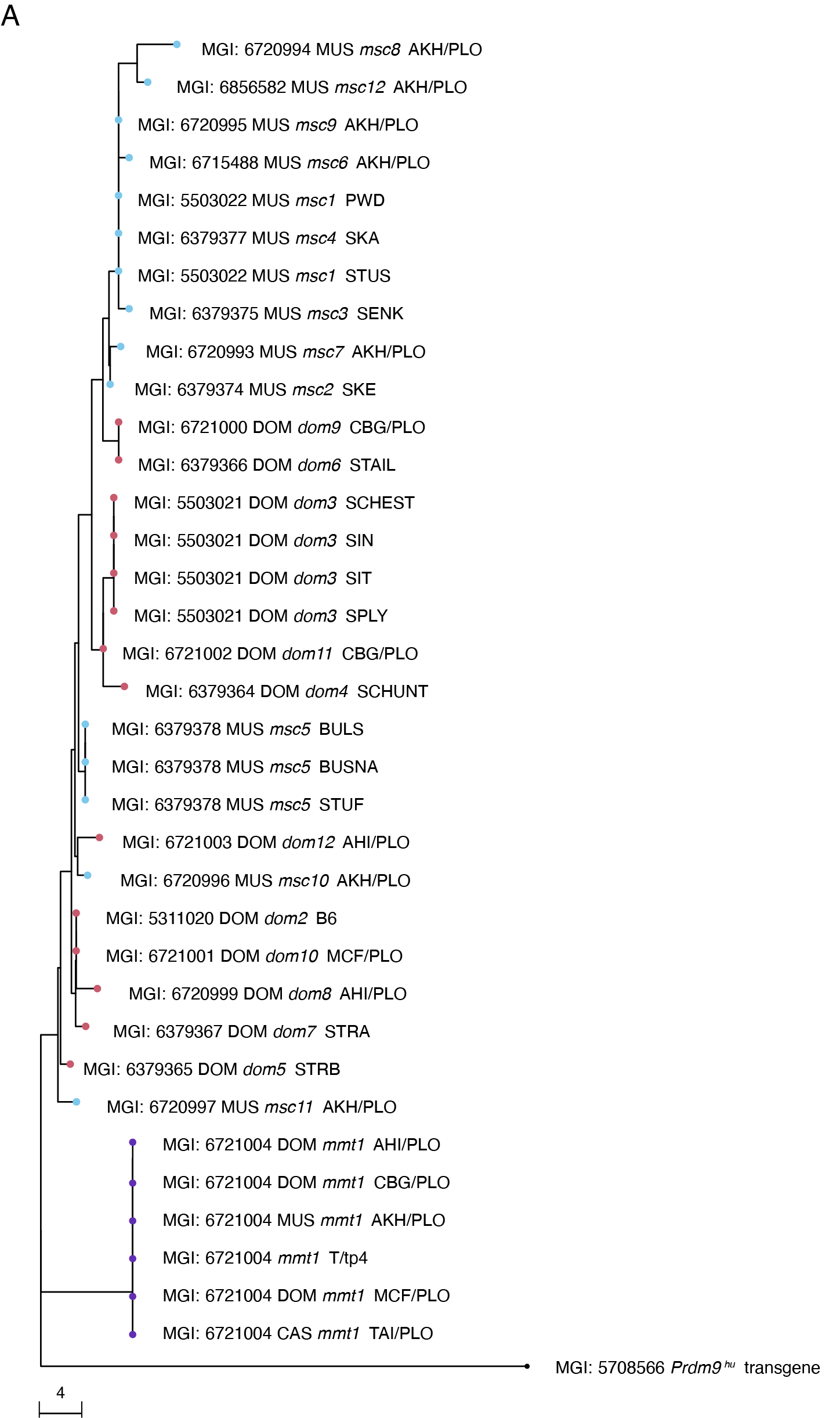


**Figure S8: Neighbor-joining tree based on Hamming-Distances of nucleotides in the Prdm9 minisatellite** calculated after removing hypervariable nucleotides that code for the amino acids responsible for DNA binding specificity of PRDM9 and that are known to be under strong positive selection.

**Table S1: Primers used to genotype the Hstx2 interval from (a)(**[**Lustyk et al. 2019**](#_ENREF_54)**) and (b) P. Jansa personal communication**

| **STR marker** | **Forward Primer: (5’ to 3’)** | **Label** | **Reverse Primer: (5' to 3')** | **position in mm10** |
| --- | --- | --- | --- | --- |
| SX65100^a^ | AAAAAGGCTGCTGGAAGTCA | 6-FAM | ATGAGGCTGGGATTCTTCCT | ChrX:65100392-65100563 |
| SR51^b^ | AAGCAGGAGTGGGTGAGTTG | 6-FAM | CTCCCTTTTGCCAAGGTTTA | ChrX:67841601-67841743 |
| SX69084^a^ | AGGCCTTCTGGGCTTATCTC | HEX | AAAGCTCATGGATGAGAAAACA | ChrX:69084171-69084417 |

**Table S2: Variation, Introgression, and Recombination rate of outcrossed populations with data from (a) (**[**Lawal et al. 2021**](#_ENREF_52)**) (b) (**[**Wooldridge and Dumont 2022**](#_ENREF_91)**) (c) (**[**Banker et al. 2022**](#_ENREF_9)**) (d) (**[**Staubach et al. 2012**](#_ENREF_78)**)**

| **outcrossed populations** | **# SNPS** | **SNP density (bp/SNP)** | **# population private variants** | **Average introgression per individual (%)** | **% of the genome affected by introgression** |
| --- | --- | --- | --- | --- | --- |
| AKH | 10,937,288^a^ | 225^b^ | 1,872,782^a^ | NA | 4.90^d^ |
| MCF | 11,108,085^a^ | 222^b^ | 2,208,483^a^ | 0.069^c^ | 2.80^d^ |
| CBG | 11,930,888^a^ | 206^b^ | 545,881^a^ | 0.068^c^ | 2.80^d^ |
| AHI | 17,877,283^a^ | 138^b^ | 3,333,440^a^ | 0.036^c^ | NA |

**Reference:**

Staubach F, Lorenc A, Messer PW, Tang K, Petrov DA, Tautz D. 2012. Genome patterns of selection and introgression of haplotypes in natural populations of the house mouse (*Mus musculus*). PLoS Genet. 8(8):e1002891. doi:10.1371/journal.pgen.1002891.

**Table S3: Variation in sperm counts (SC) grouped by Prdm9 genotype of F_1_ hybrid male offspring,** evaluated using ordinary one-way ANOVA with Bonferroni’s multiple comparisons test. Significant P-values are bold.

| **Ordinary**  **one-way ANOVA** | **Summary** | **Bonferroni Adjusted**  **P- Value** | **Mean 1** | **Mean 2** | **Mean Diff.** |
| --- | --- | --- | --- | --- | --- |
| *msc6* vs. *msc1* | ******* | **0.0001** | 22.1 | 0 | 22.1 |
| *msc7* vs. *msc1* | ******* | **0.0007** | 20.8 | 0 | 20.8 |
| *msc8* vs. *msc1* | ****** | **0.0043** | 18.2 | 0 | 18.2 |
| *msc9* vs. *msc1* | ***** | **0.0246** | 20.3 | 0 | 20.3 |
| *msc10* vs. *msc1* | ****** | **0.0088** | 18 | 0 | 18 |
| *msc11* vs. *msc1* | ns | >0.9999 | 11.4 | 0 | 11.4 |
| *msc12* vs. *msc1* | ns | >0.9999 | 9.97 | 0 | 9.97 |
| *mmt1^KH^* vs. *msc1* | ****** | **0.0029** | 18.2 | 0 | 18.2 |
|  |  |  |  |  |  |
| *msc6* vs. *msc12* | ******* | **0.0005** | 22.1 | 9.97 | 12.1 |
| *msc7* vs. *msc12* | ****** | **0.0099** | 20.8 | 9.97 | 10.8 |
| *msc8* vs. *msc12* | ns | 0.1046 | 18.2 | 9.97 | 8.26 |
| *msc9* vs. *msc12* | ns | >0.9999 | 20.3 | 9.97 | 10.3 |
| *msc10* vs. *msc12* | ns | 0.3438 | 18 | 9.97 | 8.06 |
| *msc11* vs. *msc12* | ns | >0.9999 | 11.4 | 9.97 | 1.39 |
| *mmt1^KH^* vs. *msc12* | ***** | **0.0464** | 18.2 | 9.97 | 8.28 |
|  |  |  |  |  |  |
| *mmt1^CB^* vs. *dom2* | ******** | **<0.0001** | 20.3 | 0 | 20.3 |
| *mmt1^MC^* vs. *dom2* | ns | 0.1394 | 14.3 | 0 | 14.3 |
| *mmt1^AH^* vs. *dom2* | ******** | **<0.0001** | 24.6 | 0 | 24.6 |

**Table S4: Variation in testes weight normalized by body weight (TW/BW) grouped by Prdm9 genotype of F_1_ hybrid male offspring,** evaluated using ordinary one-way ANOVA with Bonferroni’s multiple comparisons test. Significant P-values are bold.

| **Ordinary**  **one-way ANOVA** | **Summary** | **Bonferroni Adjusted**  ***P-* Value** | **Mean 1** | **Mean 2** | **Mean Diff.** |
| --- | --- | --- | --- | --- | --- |
| *msc6* vs. *msc1* | ******** | **<0.0001** | 0.928 | 0.265 | 0.663 |
| *msc7* vs. *msc1* | ******** | **<0.0001** | 0.890 | 0.265 | 0.625 |
| *msc8* vs. *msc1* | ******** | **<0.0001** | 0.899 | 0.265 | 0.634 |
| *msc9* vs. *msc1* | ******** | **<0.0001** | 0.939 | 0.265 | 0.674 |
| *msc10* vs. *msc1* | ******** | **<0.0001** | 1.000 | 0.265 | 0.735 |
| *msc11* vs. *msc1* | ******** | **<0.0001** | 0.800 | 0.265 | 0.535 |
| *msc12* vs. *msc1* | ******** | **<0.0001** | 0.707 | 0.265 | 0.442 |
| *mmt1^KH^* vs. *msc1* | ******** | **<0.0001** | 0.972 | 0.265 | 0.707 |
|  |  |  |  |  |  |
| *msc6* vs. *msc12* | ******* | **0.0004** | 0.928 | 0.707 | 0.221 |
| *msc7* vs. *msc12* | ***** | **0.0167** | 0.890 | 0.707 | 0.183 |
| *msc8* vs. *msc12* | ****** | **0.0023** | 0.899 | 0.707 | 0.192 |
| *msc9* vs. *msc12* | ns | 0.1015 | 0.939 | 0.707 | 0.232 |
| *msc10* vs. *msc12* | ******** | **<0.0001** | 1.000 | 0.707 | 0.293 |
| *msc11* vs. *msc12* | ns | >0.9999 | 0.800 | 0.707 | 0.093 |
| *mmt1^KH^* vs. *msc12* | ******** | **<0.0001** | 0.972 | 0.707 | 0.265 |
|  |  |  |  |  |  |
| *mmt1^CB^* vs. *dom2* | ******** | **<0.0001** | 0.848 | 0.310 | 0.538 |
| *mmt1^MC^* vs. *dom2* | ******** | **<0.0001** | 0.823 | 0.310 | 0.513 |
| *mmt1^AH^* vs. *dom2* | ******** | **<0.0001** | 0.771 | 0.310 | 0.461 |
