# Supplementary figures and images for: Natural variation in the zinc-finger-encoding exon of Prdm9 affects hybrid sterility phenotypes in mice
Source: Genetics. 2024 Jan 13;226(3):iyae004. doi: 10.1093/genetics/iyae004 (PMC10917509; doi:10.1093/genetics/iyae004)

## Original Sampling Sites

**Subspecies:** ● MMD ● MMM

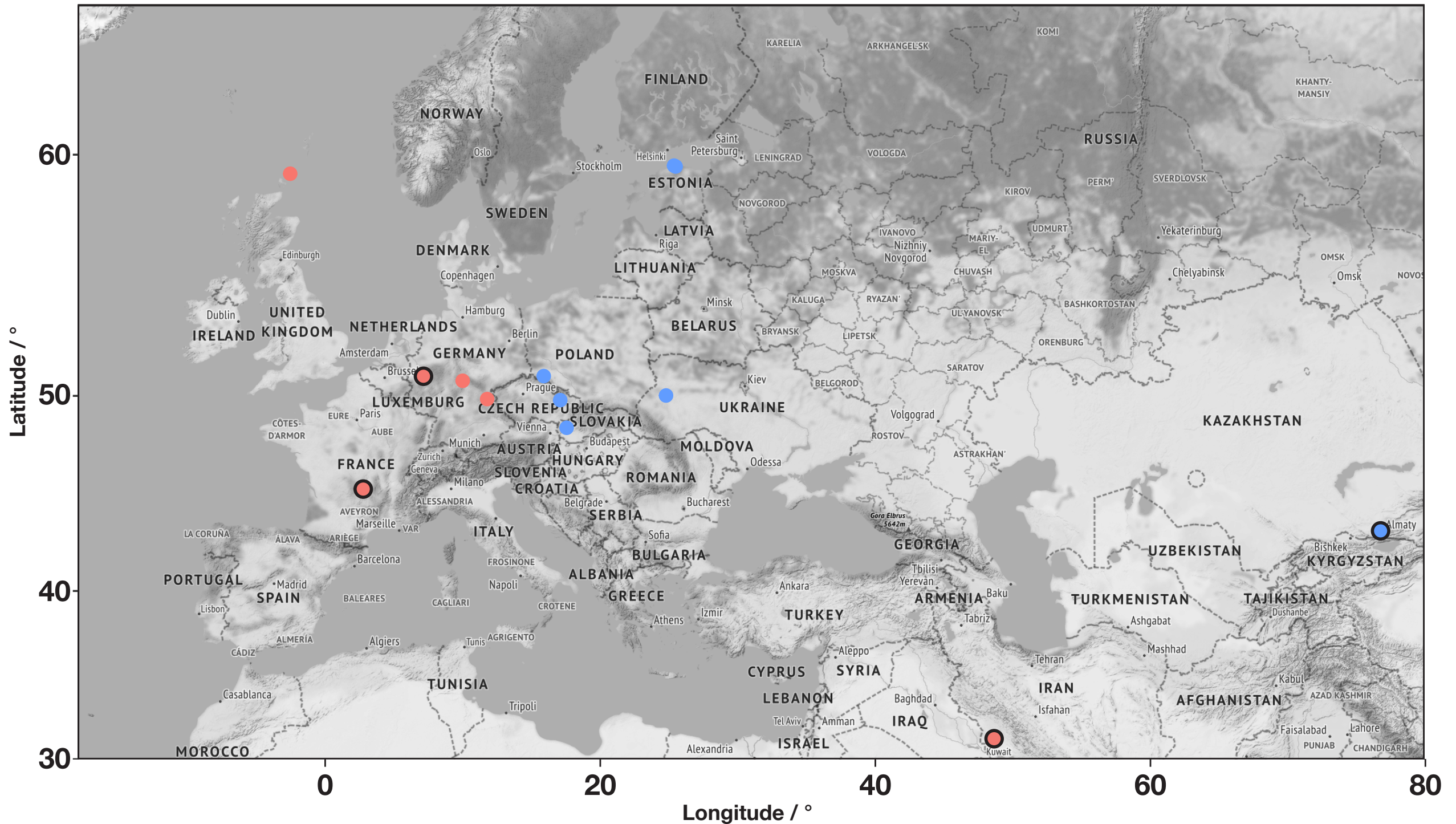

Supplement: iyae004_Supplementary_Data [file iyae004_supplementary_data.zip › Figure_S1_GENETICS-2023-306660.pdf]

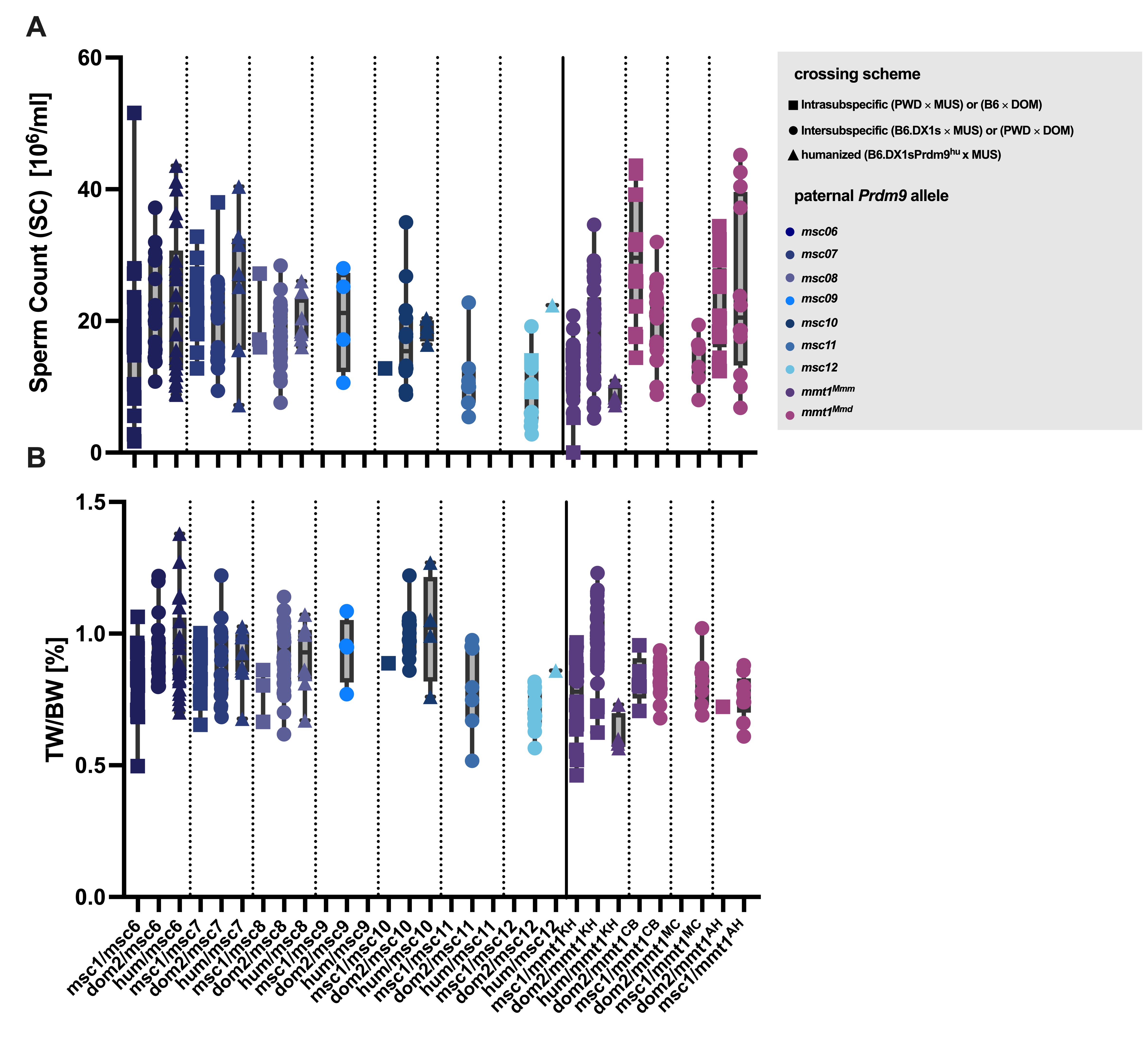

Supplement: iyae004_Supplementary_Data [file iyae004_supplementary_data.zip › Figure_S4_GENETICS-2023-306660.jpg]

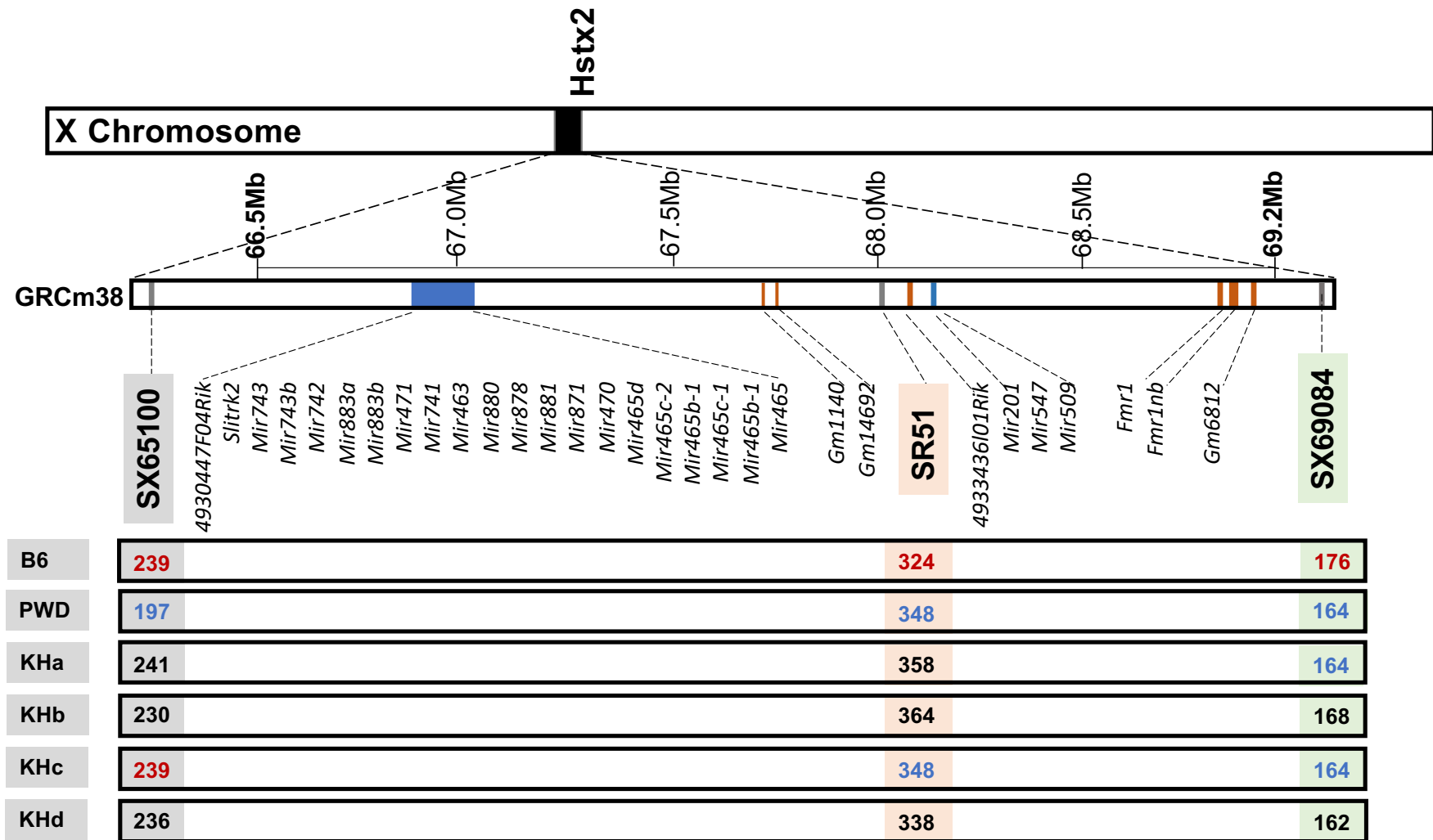

Supplement: iyae004_Supplementary_Data [file iyae004_supplementary_data.zip › Figure_S6_GENETICS-2023-306660.pdf]

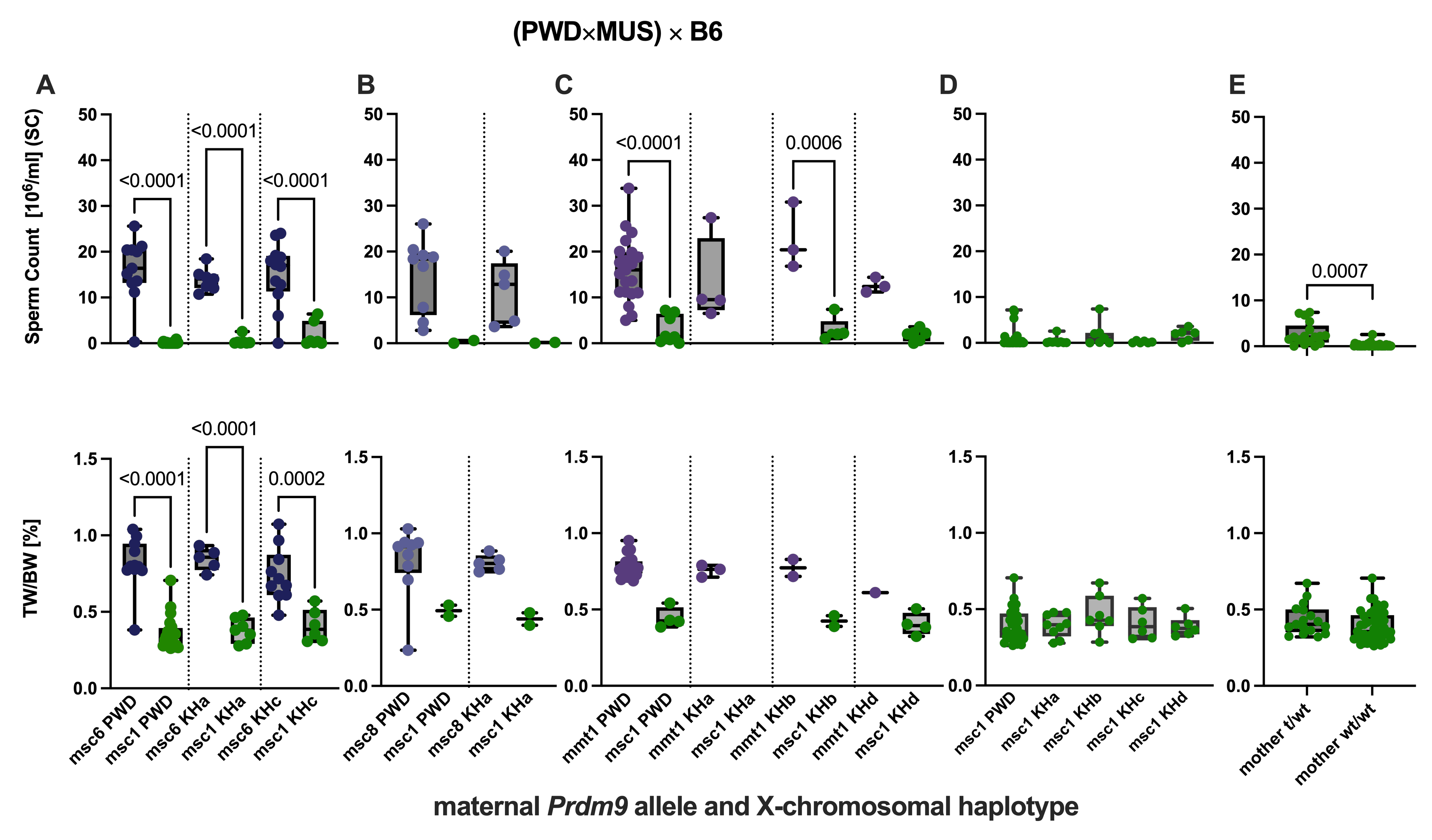

Supplement: iyae004_Supplementary_Data [file iyae004_supplementary_data.zip › Figure_S7_GENETICS-2023-306660.tif]

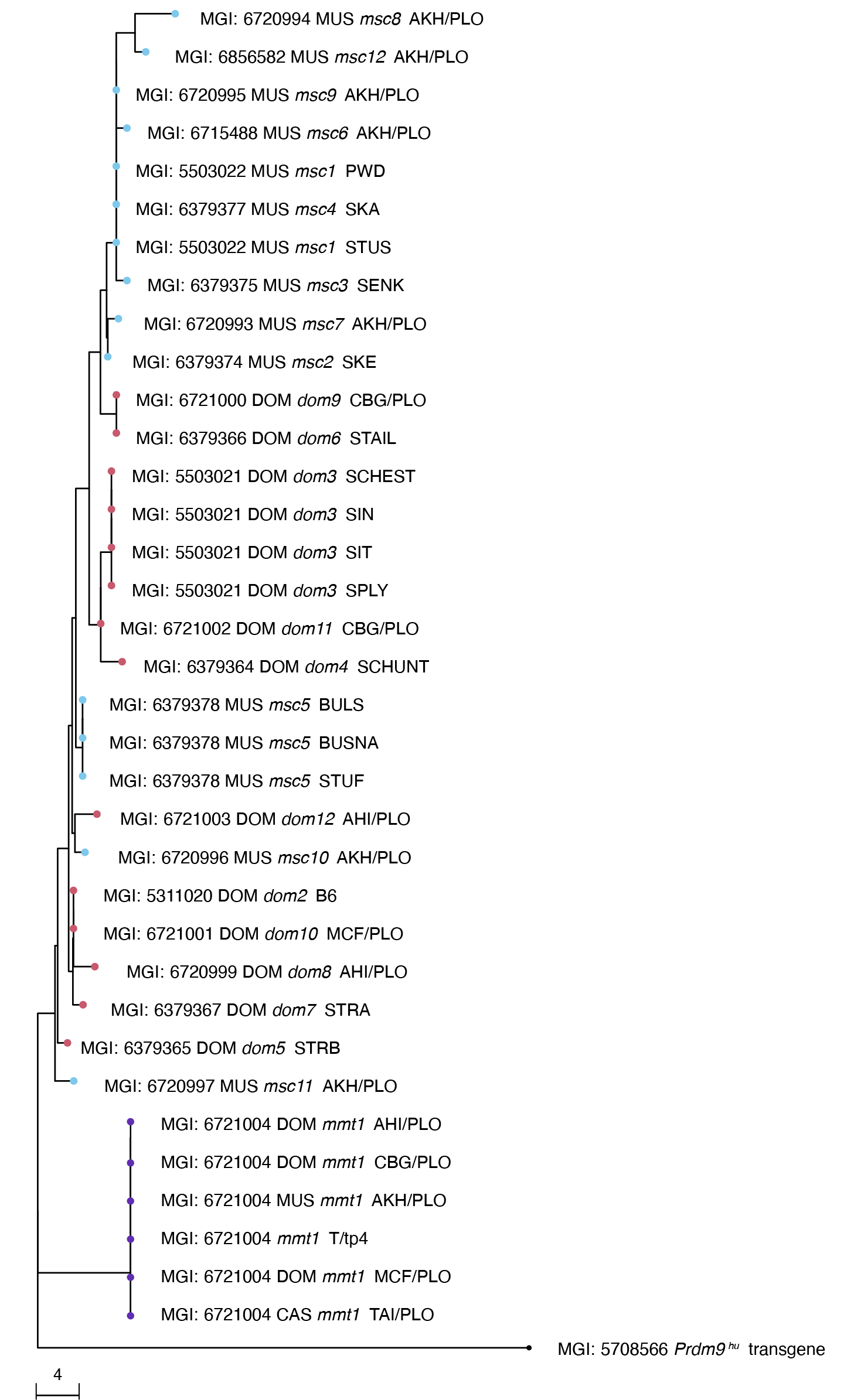

Supplement: iyae004_Supplementary_Data [file iyae004_supplementary_data.zip › Figure_S8_GENETICS-2023-306660.png]
